# Hypnotizability and Placebo Analgesia in Waking and Hypnosis as Modulators of Auditory Startle Responses in Healthy Women: An ERP Study

**DOI:** 10.1371/journal.pone.0159135

**Published:** 2016-08-03

**Authors:** Vilfredo De Pascalis, Paolo Scacchia

**Affiliations:** La Sapienza University of Rome, Rome, Italy; Vanderbilt University, UNITED STATES

## Abstract

We evaluated the influence of hypnotizability, pain expectation, placebo analgesia in waking and hypnosis on tonic pain relief. We also investigated how placebo analgesia affects somatic responses (eye blink) and N100 and P200 waves of event-related potentials (ERPs) elicited by auditory startle probes. Although expectation plays an important role in placebo and hypnotic analgesia, the neural mechanisms underlying these treatments are still poorly understood. We used the cold cup test (CCT) to induce tonic pain in 53 healthy women. Placebo analgesia was initially produced by manipulation, in which the intensity of pain induced by the CCT was surreptitiously reduced after the administration of a sham analgesic cream. Participants were then tested in waking and hypnosis under three treatments: (1) resting (Baseline); (2) CCT-alone (Pain); and (3) CCT plus placebo cream for pain relief (Placebo). For each painful treatment, we assessed pain and distress ratings, eye blink responses, N100 and P200 amplitudes. We used LORETA analysis of N100 and P200 waves, as elicited by auditory startle, to identify cortical regions sensitive to pain reduction through placebo and hypnotic analgesia. Higher pain expectation was associated with higher pain reductions. In highly hypnotizable participants placebo treatment produced significant reductions of pain and distress perception in both waking and hypnosis condition. P200 wave, during placebo analgesia, was larger in the frontal left hemisphere while placebo analgesia, during hypnosis, involved the activity of the left hemisphere including the occipital region. These findings demonstrate that hypnosis and placebo analgesia are different processes of top-down regulation. Pain reduction was associated with larger EMG startle amplitudes, N100 and P200 responses, and enhanced activity within the frontal, parietal, and anterior and posterior cingulate gyres. LORETA results showed that placebo analgesia modulated pain-responsive areas known to reflect the ongoing pain experience.

## Introduction

Human pain is a potent stressor with immediate relevance to survival and is believed to be a multi-faceted protective experience involving the activity of sensory-discriminative, affective-emotional, attentional-cognitive, behavioral and social learning systems [1–3]. Multiple psychological factors have been suggested to modulate the magnitude of pain, i.e., past experience [4, 5], classical conditioning [6–13], suggestibility [14, 15], expectancy [16], and ritualistic therapeutic acts [14, 17, 18]. This type of modulation is believed to happen through a top-down neurophysiological process that, when activated by one or more of the above mentioned factors, may produce neurophysiological changes that affect pain perception. The use of placebos and hypnosis represents two ways of how the top-down control can modulate pain perception, but the research of placebo and hypnosis often follows two separate tracks and their neurobiological similarities and differences are yet not fully understood. We have known, for a long time, that hypnotic analgesia is not a variant on placebo analgesia since highly hypnotizable individuals report feeling less pain during hypnosis than during placebo condition [19], suggesting that the effects of placebo and hypnosis analgesia are at least in part separate processes. A recent literature review on brain activity changes to placebo and hypnotic analgesia has highlighted similarities and differences between these two treatments [20]. First, these treatments produce similar changes in the activity of a number of brain regions labeled as pain network (i.e., somatosensory cortex, ACC, insula, thalamus, and prefrontal cortex). The activation of prefrontal cortex is necessary for cognitive evaluation and plays a leading role during both placebo and hypnotic analgesia treatments [21–24]. Pain relief elicited through these treatments are mediated by dopaminergic activity in the prefrontal cortex [25–28] and caused by changes in expectation [29, 30]. Second, the major differences between these two treatments lie in the fact that placebo analgesia produces functional changes in several parts of the limbic system (i.e., amygdala, hypothalamus and hippocampus, periaqueductal gray, nucleus accumbens). In contrast, hypnosis causes changes of activity in the occipital areas which are elicited by mental imagery [31–33] and basal ganglia which operate in the voluntary movement regulation [32, 34, 35].

The startle response is a fast defensive mechanism consisting of sequential sudden contractions of somatic muscles and is usually evoked by acoustic stimuli, with the likely purpose of facilitating the flight reaction and/or protecting the body from sudden attack. It has been proved that positive emotional states attenuate/inhibit defensive reflexes and that negative states enlarge/facilitate defensive reflexes [36]. Such affective startle modulation has been explained in terms of motivational priming: aversive emotional stimuli prime the defensive motivational system and, thereby, facilitate defensive reflexes, whereas appetitive emotional stimuli inhibit defensive reflexes [36]. Although startle responses are muscular and event-related potentials (ERPs) are neural, research has demonstrated that N100 and P200 waves of the ERPs can be considered electrocortical indicators of the startle response [37–39], but little effort has been devoted to parallel EMG startle with ERP responses [40]. Startle responses to pulse-alone stimuli and ERP responses are also sensitive to arousal and affective states [41–44]. The N100 wave of the ERPs reflects the processing of the auditory stimulus’s physical features (e.g., the intensity) [45], and, therefore, is a measure of the initial registration and attribute selection of an auditory stimulus [46]. The P200 wave of the ERP is thought to represent a later stage of stimulus processing and is viewed as an index of both sensory processing and cognitive demands as the stimulus classification process [47]. Thus is not surprising that many studies have shown that negatively charged stimuli elicit larger eye-blink reflexes than positively ones [48–51] and that the N100 and P200 waves of the ERPs to auditory startles are sensitive to emotional and arousal components of the stimulation. [44]. Negative and positive emotional stimuli usually elicit larger N100 and P200 amplitudes than neutral ones [52] in participants highly sensitive to punishment [53].

Research has shown that eye-blink startle response is modulated by the attention devoted to the startle probe [54] or engagement in a mentally demanding task [55]. For example, the hand blink reflex has been recently found of significantly greater magnitude when the stimulated hand is near to the face than when it is far [56]. These findings demonstrate that brainstem activities mediating defensive reflexes can receive a top-down modulation to select adequately the external potential threats. Moreover, factors such as expectation [57] and hypnotizability can affect both pain sensation and startle responses [58–62]. Although research has demonstrated that fear-evoking stimuli consistently potentiate the magnitude of the blink reflex [36, 63–66], studies investigating the pain potentiation of the startle response has produced only inconclusive results. Some of them have reported no pain potentiation of the startle response, regardless of the stimulus intensity and the subjects’ sensitivity to startle modulation [67, 68]. Research using the classic Cold Pressor Test (CPT) has also produced inconclusive results [69, 70]. One study has reported a reduction in startle magnitude [71], another study has shown a significant potentiation of the startle response [72], and a more recent study has observed no significant effects [73]. Thus, in the present study, to disentangle these contrasting results, we evaluated how tonic cold pain can influence motor and ERP components of the auditory startle response (ASR). Furthermore, hypnotic analgesia is one of the most reliable hypnotic phenomena [61, 74–78] and, although the influence of cognition and attention in placebo and hypnosis analgesia have been demonstrated, the neural mechanisms underlying to these two treatments are still poorly understood. Therefore, aim of the present study was an attempt to evaluate how hypnotic susceptibility, pain expectation, hypnosis and placebo treatments can influence tonic pain relief and how this effect is reflected on eye-blink and ERP components of the ASR. To induce tonic pain we used the cold cup test (CCT) [79], requiring to hold in the right hand an iced water plastic cup at -10°C.

In the present study placebo analgesia was initially produced by a manipulation procedure in which participants were simply fooled into thinking that a sham “analgesic cream” was working to reduce their pain perception. The noxious-stimulus strength was then restored to its original level and delivered during a placebo cream treatment (Placebo) and a painful cream-free intervention (Pain), with a pre-stress Baseline serving as a control.

On the basis of our previous hypnotizability startle findings [60], we expected that hypnotizability and pain reduction should be associated with enhanced inhibitory responses, i.e., smaller ASRs, and smaller N100 and P200 ERP waves. Mainly, we expected that high hypnotizable (HH) participants, during the Baseline and Placebo, as compared to the Pain, should exhibit greater reductions of pain and distress scores with smaller ASRs and ERP amplitudes. A further aim of the present study was to evaluate: (1) how placebo treatment during waking may differ from placebo treatment during hypnosis; (2) how individual differences in pain expectation and hypnotizability may affect the reduction of pain, and how this response may modulate the magnitude of ASR and ERP responses.

Recent work has demonstrated that low-resolution brain electromagnetic tomography (LORETA) is a valid tool for relevant pain research [31, 80]. Thus, a further aim of the present study was to use LORETA analysis of auditory ERP waves to identify the influence of pain reduction, hypnotizability, and pain expectation, in the activity of cortical regions involved in auditory-startle processing.

## Materials and Methods

### Ethics statement

The research was conducted according to the ethical standards of the American Psychological Association (APA). Approval of this study was obtained from the Ethics Committee of the Department of Psychology, La Sapienza University of Rome, Italy, on December 2, 2011. All the participants gave their written consent to participate in the study, in accordance with the Declaration of Helsinki.

### Participants and measures of hypnotizability

Fifty-eight right-handed women volunteers were recruited through university courses by advertisements. They were all new to hypnosis experience with the exception of one participant who had experienced hypnosis one year before. Handedness was measured by the Italian version of the Edinburgh Inventory Questionnaire [81, 82]. We included only healthy participants free of medication who had no history of psychiatric or neurological disorders or any lifetime history of hearing problems. The subjects were asked to refrain from smoking or drinking coffee for at least three hours before the EEG recording. Only 53 women (M = 23, SD = 1.5 yr, range 18–27 yr) participated in the study, since 5 participants were excluded for large eye-blink and movements artifacts in the EEG record. The study consisted of two sessions. The first session consisted in the administration of the Italian version of the Stanford Hypnotic Susceptibility Scale, Form C (SHSS:C, N = 53, M = 6.3, SD = 3.4; Md = 6.0) [83] to measure the hypnotizability level of each participant. The second session (2 p.m. to 3 p.m.) consisted of a pain manipulation treatment (to enhance placebo effect), followed by three treatments administered in both waking and hypnosis condition. Hypnosis was induced using the Stanford Hypnotic Clinical Scale (SHCS; N = 53, M = 2.4, SD = 1.7; Md = 2.0) [84] during which electrophysiological recordings were obtained. Since the level of hypnotizability of each subject (i.e., low, medium, or high hypnotizability) did not change across the SHSS:C and SHCS measures, only the measure of the SHSS:C was used for statistical analyses. More details for participant's selection are reported in the Section A in S1 Appendix.

### Pain manipulation, treatments and pain/distress ratings

**Fig 1:**
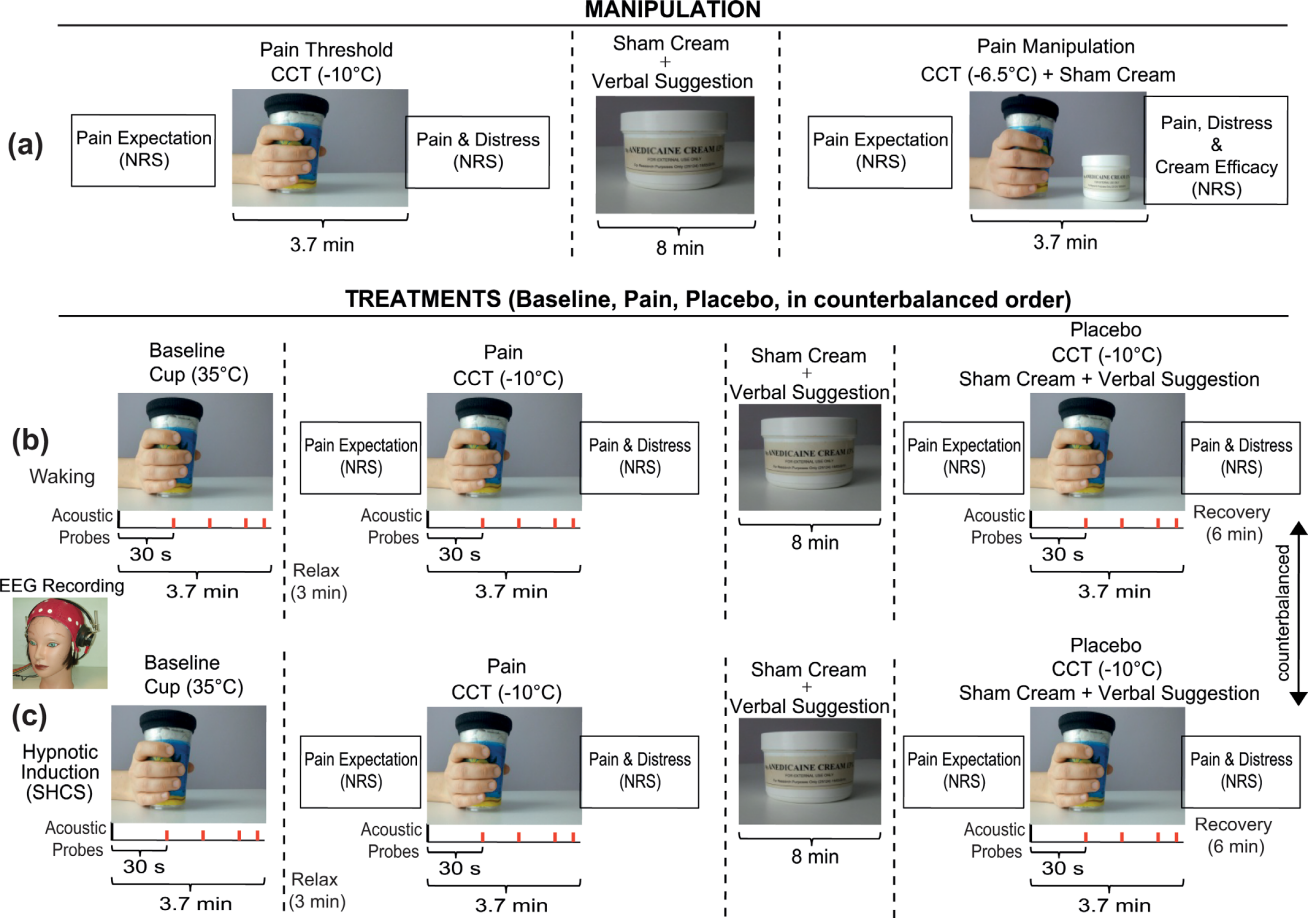
Schematic representation of experimental design and procedure. Panel (a) displays Manipulation procedure including the initial Pain Expectation rating, the measure of Pain Threshold, the administration of Sham Cream plus Verbal Suggestion and Pain Manipulation. In panel (b) are shown the Baseline, Pain and Placebo treatments in waking condition. In panel (c) are shown the same treatments administered after the hypnotic induction (Stanford Hypnotic Clinical Scale, SHCS). Treatments, within each participant, were administered in the same order in waking and hypnosis condition, but both conditions and treatments were counterbalanced across participants. Before administration of pain and placebo treatment, participants rated the level of pain expectation. Following each pain treatment, participants rated the intensity of experienced pain and distress sensation. In panels (b) and (c), for each treatment, are displayed acoustic probes (115 dB, 40 ms) delivered to elicit startle blink responses.

Fig 1 depicts a schematic representation of the second experimental session. Pain manipulation phase consisted in the administration of the CCT for two times. Before of the first administration of the CCT participants were required first to rate their initial pain expectation for the whole experimental testing (Pain Expectation) and then to hold, in their right hand, a thin plastic cup full of frozen water at -10°C. This procedure served to measure pain threshold and, for participants, to form a first experience of pain and distress sensation (Section B in S1 Appendix). During the second CCT administration participants were deceived into thinking that a sham analgesic cream (“Anedicaine Cream 13%”), smeared on the palm of their right hand, was working to reduce their pain sensation [7, 12, 16, 85, 86] (Section C in S1 Appendix). They were first informed that the "Anedicaine Cream" would have had its maximum effect after eight minutes from the administration, and that the effect would be lost completely after 20 minutes. During the application of the cream to the surface of the palm and fingers of the right hand, the participants were given a suggestion devoted to induce a reduction of pain sensation (see Fig 1A and Section C in S1 Appendix). Eight minutes after the application of the cream, participants were required first to rate their pain expectation and, then, to tightly hold a frozen water cup for 3.7 minutes. At this point the temperature of the plastic cup was reduced from -10°C to -6.5°C. Just after this manipulation, participants first rated their experienced pain and distress and then the level of cream efficacy (NRS). The manipulation procedure led participants to report a reduction of both pain and distress scores on the NRS (Pain: M = 23.2, SD = 12.1, t = 9.7, p < 0.0001; Distress: M = 11.7, SD = 11.3, t = 6.4, p < 0.0001). The electrodes were then attached to the participants for electrophysiological recordings. During waking, and following the hypnotic induction (SHCS), each participant was subjected to the following three separate treatments: (1) Baseline, no-painful/no-treatment, i.e., holding in the right hand a cup full with a mixture of wax and iron filings at a temperature near body level of about 35°C; (2) CCT, i.e., holding a cold cup at -10°C in the right hand [79] without any cream administration (Pain); and (3) CCT plus sham cream with suggestion treatment (Section C in S1 Appendix) for pain relief (Placebo). Somatic (eye blink) and ERP responses to acoustic startle probes were elicited during the Baseline, Pain, and Placebo treatments in waking and hypnosis condition. Before each painful treatment, participants rated their level of pain expectation (NRS), and after each treatment they rated the level of the experienced pain and distress (NRS, Fig 1B and 1C). The sequence of waking and hypnosis conditions, as well as the order of presentation of the three treatments, was counterbalanced across participants. However, within each participant, the order of the three treatments was held the same for both waking and hypnosis conditions. The whole of the testing took approximately 11 min, 3.7 min for the Baseline and 3.7 min for the Pain and Placebo treatments. In addition, an inter-treatment relaxing interval of 3 min was given after each treatment. Following the administration of the “Anedicaine Cream" a time period of 8 min was given to ensure an effect of the pharmacological treatment and then the CCT was administrated (3.7 min). A recovery period of 6 min was given between waking and hypnosis conditions. Before starting the hypnotic induction, participants were required to rate their expectation and motivation to experience hypnosis using two separate NRS scales. Following the hypnotic induction and hypnotic testing, paticipants were administered a fractionation procedure (reverse counting from 20 to 1) devoted to enhance hypnosis depth [87]. After this procedure participants rated their hypnotic depth on a NRS scale.

### Sham analgesic cream

The sham analgesic cream was a simple yellow and scented moisturizer. The smell was obtained by mixing 5 drops of essential oil (tea tree oil) into 300 grams of cream. Participants were told: “In this second session, we are interested in exploring the subjective reactions to painful stimuli in both waking and hypnosis conditions. This study is part of an international collaborative research study devoted to compare the pain reduction effects of a known analgesic cream on brain activity during intense acoustic stimulations. The cream consists of an active pharmacological natural compound that has been prepared in our university hospital and proved to have strong analgesic effects free from side effects. In this experiment, we are following a double-blind procedure that includes a sham cream as a control and, for this reason, neither the experimenter nor the participants are aware of which ointment will be used.” We used this experimental protocol to avoid the "analgesic cream" being seen as a sham treatment, since the participants were all students in psychology courses. In this way, we tried to avoid any surprise effect on the startle and ERP responses [88]. Although, in this way, we may have reduced the placebo effect, we avoided bias effects caused by possible suspicions that the cream could be a sham drug [89, 90]. After this introduction, participants were required to fill out the consent form and a state anxiety questionnaire (STAI-Y1, State-Trait Anxiety Inventory by Spielberger and colleagues [91]; Cronbach’s α for the STAI-Y1 in the present research was .85). Participants were informed about the difference between pain and distress sensation and then they were required to rate their pain expectation on a 0 to 100 ‘Numeric Rating Scale’ (NRS) [92].

### Acoustic startle stimuli

Acoustic startle stimuli were binaurally presented through headphones (Telephonics) and produced using the Wavelab-5.0 software. The acoustic startle stimulation consisted of three trial blocks. Each block included the presentation of 24 acoustic stimuli and began with a 2 min adaptation period consisting of 70 dB broadband noise (0–44 kHz), which continued as background noise throughout the session. Of the 24 acoustic stimuli, 10 were pulse-alone (PA) stimuli and 14 were pairs of acoustic stimuli, a prepulse followed by a pulse with a lead interval of 120 ms. In order to avoid habituation, i.e., reduced responding across stimulus type, PA and prepulse-pulse (PP) stimuli were presented in pseudorandom order to ensure that there were never more than two PA or PP in succession [93]. The interval between the PA and PP stimuli varied between 7–10 seconds. PA startle stimuli were binaural acoustic white-noise probes (115 dB, 40 ms duration, instantaneous rise time < 1 ms). PP stimulation consisted of two white noise pulses, the prepulse (85 dB, 20 ms duration) and the pulse (115 dB, 40 ms), with a lead interval between the onset of the prepulse and the onset of the pulse stimuli of 120 ms. The stimulus had an almost instantaneous rise time (< 1 ms). Since the CCT has a strong negative valence, which is known to increase over the course of time [94], in each trial block we began to deliver the acoustic stimuli 30 s after the painful stimulation had started. Before starting the EEG recording, additional four PA and four PP stimuli were delivered superimposed to background noise. These served as habituation trials (1.5 min) and were not included in further analysis. Initial startle responses are usually exaggerated in size. After a few trials, habituation follows a more gradual course. Therefore it is common to exclude initial trials from further analysis (‘‘habituation trials”) [93]. During the three treatments, participants were asked to look straight ahead to a fixation point in the center of a 15-inch monitor.

Clinical studies that have used a prepulse of 85 dB and a 70 dB background noise (i.e., providing a signal-to-noise ratio of +15 dB) have shown that startle and prepulse inhibition responses are sensitive indexes of individual differences in schizophrenia [95–97] and anxiety spectrum diseases [98]. This method has been also found valid to detect individual differences in anxiety [99] and hypnotic susceptibility [60] in nonclinical participants. When background noise is not used, the prepulse and the pulses may be so salient that individual differences in startle and prepulse inhibition do not appear in clinical and normal populations [100].

The ERP responses elicited by the startle probe of the prepulse-startle pair (lead interval of 120 ms) were partially overlapping with the ERPs elicited by the preceding prepulse. Thus, we decided that the detection and measure the N100 and P200 waves must be done by visual inspection of each complex waveform. Thus, due to the complexity of this scoring process prepulse-pulse inhibition responses will be analyzed and reported later in another paper.

### EEG/EMG recordings

EEG, EMG, and electro-ocular (EOG) activities were acquired using a 40-channel NuAmps DC amplifier system (Neuroscan Inc.), set at a gain of 200, a sampling rate of 1000 Hz, and with signals band-limited to 500 Hz. Data were recorded and stored on a computer running Neuroscan Acquire 4.3 software. Electrode impedance was lower than 5 kΩ. The horizontal EOG was monitored via a pair of tin electrodes placed 1 cm lateral to the outer canthus of each eye, while the vertical EOG was monitored via a separate bipolar montage placed above and below the center of the left eye.

Electrodes for EMG recording of the muscle orbicularis oculi were attached below the participant’s right eye at an inter-electrode distance of 1.5 cm. EEG data were recorded from 30 scalp sites (Fp1, Fp2, F7, F8, F3, F4, FT7, FT8, T3, T4, FC3, FC4, C3, C4, CP3, CP4, TP7, TP8, T5, T6, P3, P4, O1, O2, Fz, FCz, Cz, CPz, Pz, Oz) using a pure-tin electrode electro-cap and were referenced to digitally linked ears (A1 + A2)/2 by Neuroscan Acquire setting. The ground electrode was located 10 mm anterior to Fz. During the EEG recording, each signal was first online filtered using 50 Hz notch filter. The EEG was then reconstructed into discrete, single-trial epochs. For each stimulus, an EEG epoch length of 700-ms was used with a 200-ms pre-stimulus baseline and a 500-ms time window following the acoustic stimulus onset. Epochs were rejected from averaging if the amplitude exceeded ±75 μV, and eye blinks were corrected using Gratton et al.’s procedure [101]. Additional movement artifacts were removed manually. After removing artifacts, only responses to PA stimuli that used 6 or more epochs were accepted for PA averaging in the present study [102].

### Startle and ERP measures

The EMG signal was off-line filtered by a 28 Hz high-pass filter [103] using the Brain Vision Analyzer 2.1.0. The raw signal was then rectified and integrated online with a time constant of 10 ms [104]. The ASR was defined as the difference between the peak and baseline signal. EMG signal was epoched within a 500 ms time window with a baseline of 200 ms prior to the onset of the startle stimulus. Data were baseline corrected, visually inspected for artifact rejection, rectified, and enveloped. A time window of 40–150 ms was used to detect ASRs. The occurrence of a startle was considered when there was at least an EMG value greater than 3 SD above a 200 ms baseline mean (for details see [105]). Peak latency was defined as the point of maximal amplitude occurring within a time window of 40–150 ms after the pulse stimulus onset [93]. Each response was manually confirmed and participants with more than 4 non-responses were excluded from the study, according to the criteria of Braff and collaborators [106].

All EEG-averaged signals were digitally filtered (48-Hz low pass) and baseline corrected.

After baseline correction, two reliable ERP components, mainly pronounced at the central recording site (Cz), were identified. The first component was the N100 (112.3 ± 9.8 ms), which was quantified at Cz as the baseline-to-peak difference in voltage for the most negative peak within a window of 80–150 ms following the startle tone onset. The second component was the positive P200 (197.4 ± 18.4 ms), which was quantified at Cz, as the most positive peak in a window of 165–240 ms.

### LORETA source localization analysis

The LORETA-KEY software package (version v20150415 by R. D. Pascual-Marqui, The KEY Institute for Brain-Mind Research, Zurich, Switzerland; https://www.uzh.ch/keyinst/loreta) was used for source localization analysis of the ERP responses. This method enables the spatial identification and analysis of brain cortical activity via conventional EEG recordings [107–110] and has been found useful for the analysis of different time segments of ERPs [80, 111, 112]. LORETA computes current density (μA/mm^2^, i.e., the amount of electrical current flowing through a solid) without assuming any number of active sources [113]. The LORETA solution space (i.e., the locations in which sources can be found) is composed of 6239 cubic elements (“voxels,” 5 mm^3^) and is limited to cortical gray matter and hippocampi, as defined by a digitized MRI available from the Montreal Neurologic Institute (MNI; Montreal, Quebec, Canada) [114–119]. Before performing LORETA analyses, the EEG was resampled at 256 Hz.

LORETA source localization was calculated using coordinates of the 30 electrode positions for every subject at the mean N100 and P200 peaks for the PA trials. For wave source reconstructions and to detect differences in source activity, the subtractions of ERP traces between LH and HH and between high vs. low pain expectation participants were assessed using LORETA using time intervals of 80–120 ms and 170–210 ms, respectively, for the N100 and P200 waves. It is important to note that this localization is not a complete listing of all significantly different cortical areas, but is a listing of the local maxima of these differences.

### Statistical analyses

The analysis of the contribution of hypnotizability and rating measures to pain intensity and distress reduction was conducted in two steps. In the first step, zero-order correlations were obtained between hypnotizability (SHSS:C), self-rating measures of hypnotic depth, initial pain expectation, cream efficacy, and expectation and motivation to hypnosis versus pain/distress reductions (obtained by subtracting pain/distress rated for placebo from that rated for pain treatment) during waking and hypnosis. The significance of these correlations was assessed by using the bias-corrected bootstrap method, which is effective in controlling for type 1 errors associated with multiple comparisons [120]. This bootstrap analysis was performed in two steps. In the first step, 5000 new samples were generated by random re-sampling with replacements from the available data under the condition that each of the 5000 samples had the same size as the original. In each sample, we first computed zero-order correlations of each variable of interest separately within pain and distress reductions. Critical values for the upper and lower 95% bias-corrected confidence limits for all the zero-order correlation coefficients were then estimated. All coefficients with an associated confidence interval that did not include zero were considered statistically significant (p < 0.05).

Separate ANCOVAs (glm procedure, SAS 9.2) were then performed to test the effect of experimental manipulation on pain and distress scores wherein Hypnotizability, Pain Expectation, and Cream Efficacy were included as continuous between-subjects factors (covariates), and Treatment (Pain, Placebo) and Condition (Waking, Hypnosis) as within-subjects factors.

EMG startle amplitude measures was analyzed using a repeated measures ANCOVA with treatment (Baseline, Pain, Placebo) as within-subjects factors and Hypnotizability, Pain Reduction level, and Pain Expectation as continuous between-subjects factors (covariates). N100 and P200 peak amplitude measures were separately analyzed by using a similar ANCOVA design that focused on 4 midline recording sites (i.e., frontal, central, parietal, and occipital sites), with hypnotizability, Pain reduction level, and Pain expectation as continuous between-subjects factors.The Huynh–Feldt epsilon correction of significance levels was applied when necessary [121]. Post-hoc contrasts analyses were used when necessary (α = .05). As a graphic illustration of the direction for the main or interaction effects involving Pain Reduction Hypnotizability, or Pain Expectation on dependent measures of interest, data were grouped for the significant factor and displayed if necessary. A median split was used to form groups with high and low levels of hypnotizability (HH and LH; Md = 6.0). A similar method was used to group high and low levels of Pain Expectation (Md = 50.0) and Pain Reduction (Md = 5.0).

## Results

### Manipulation: Pain expectation versus Pain and Distress ratings

In the first administration of CCT (Fig 1A), the relation between pain expectation and pain threshold was not significant (r = 0.102, p > 0.05, Bootstrap: 95%, CI -.06 to .27). Moreover, pain expectation was highly correlated with pain perception, but not significantly with distress (pain: r = 0.50, p < 0.0001; Bootstrap: 95%, CI .35 to .62; distress: r = 0.254, p > .05, Bootstrap: 95%, CI .08 to .43). In the second CCT administration (manipulation phase), the correlation between pain expectation and pain perception was quite reduced, although it remained significant (pain: r = 0.28, p < 0.05, Bootstrap: 95%, CI .12 to .44), but these measures for distress were not significant (distress: r = 0.180, p > .05, Bootstrap: 95%, CI -.01 to .34). In addition, as expected, the correlation between cream efficacy and pain perception was significant and negative (pain: r = -0.40, p < 0.01; Bootstrap: 95%, CI .32 to .58).

### Hypnotizability and rating measures

Descriptive statistics and Pearson’s correlations, along with their 95% associated bootstrapped confidence intervals, of pain/distress reductions during waking and hypnosis (i.e., calculated by subtracting ratings/distress scores obtained during Placebo from those during Pain treatment), hypnotizability, pain expectation, hypnosis depth, cream efficay, expectation and motivation to experience hypnosis, are presented in Table 1. Pain expectation, as well as hypnotizability and cream efficacy, were significantly correlated with both pain and distress reductions during Placebo treatment in waking condition, but these correlations did not reached the significance level during hypnosis (Table 1).

**Table 1:** Correlation (N = 53) of Pain and Distress reductions (Pain minus Placebo treatment) during Waking and Hypnosis with SHSS:C, Pain Expectation, Cream Efficacy, Expectation and Motivation to Hypnosis rating scores. Means (M) and Standard Deviations (SD) for each variable are reported in the bottom and right of the table.

	Pain Reduction		Distress Reduction		
Variable	Waking	Hypnosis	Waking	Hypnosis	Mean and SD
**SHSS:C**	0.29*	0.20	0.28*	0.17	M = 6.32; SD = 3.41
**95%CI**	(0.08, 0.46)	(-0.05, 0.38)	(0.08, 0.45)	(-0.01, 0.33)	-
**Pain Expectation**	0.45†	0.19	0.38**	0.05	M = 52.64; SD = 21.70
**95%CI**	(0.24, 0.59)	(-0.01, 0.38)	(0.11, 0.54)	(-0.17, 0.26)	-
**Hypnosis Depth**	0.25	0.19	0.13	0.38**	M = 60.47; SD = 20.32
**95%CI**	(-.03, .39)	(-.04, .37)	(-0.07, 0.36)	(0.23, 0.50)	-
**Cream Efficacy**	0.38**	0.15	0.27*	0.02	M = 45.23; SD = 31.96
**95%CI**	(0.19, 0.54)	(-0.04, 0.34)	(0.11, 0.39)	(-0.18, 0.26)	-
**Expectation to Hypnosis**	0.31*	0.32*	0.27*	0.22	M = 62.89; SD = 20.32
**95%CI**	(0.16, 0.41)	(0.14, 0.44)	(0.12, 0.39)	(-0.00, 0.36)	-
**Motivation to Hypnosis**	0.26	0.13	0.26	-0.03	M = 82.83; SD = 17.25
**95%CI**	(-0.05, 0.38)	(-0.13, 0.30)	(-0.01, 0.34)	(-0.21, 0.13)	-
**Mean**	9.06	6.62	6.60	3.62	-
**SD**	14.60	14.61	2.08	11.50	-

* p < 0.05

** p < 0.01

† p < 0.001

State anxiety did not evidence any significant correlation with hypnotizability, pain expectation, and pain/distress ratings (all ps > .05).

**Fig 2:**
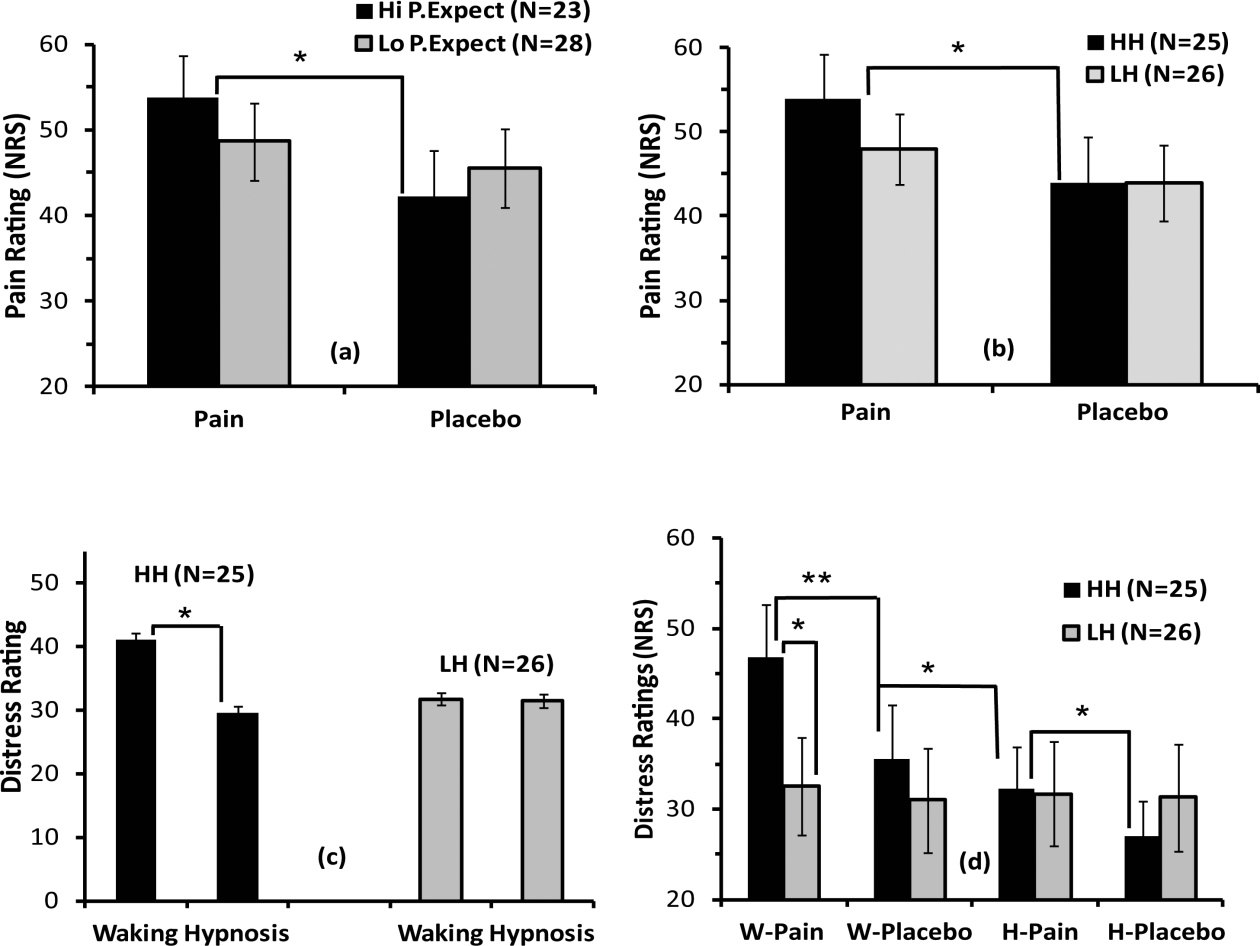
**Mean and standard errors of pain (panels a, b) and distress ratings (panels c, d), as measures of sensory-discriminative and affective-motivational components of pain.** Panel (a) displays pain scores in high and low pain expectation (Hi P.Expect and Lo P.Expect) and panel (b) in high and low hypnotizable participants (HH and LH) during pain and placebo treatments. Panel (c) shows distress ratings of waking and hypnosis conditions in high and low hypnotizable subjects (HHs and LHs). Panel (d) depicts distress ratings during pain and placebo treatments in waking and hypnosis conditions. *p < 0.05, **p < 0.01.

ANCOVA for pain intensity ratings using Hypnotizability, Pain Expectation and Cream Efficacy scores as covariates yielded a significant interaction of Treatment x Pain-Expectation (F(1,49) = 5.94, p = 0.016). Follow-up contrast analysis showed that pain rating was significantly higher for pain than placebo treatment in high pain expectation participants (p < .05, see Fig 2A).

In addition, the interaction Treatment x Hypnotizability was also significant (F(1,49) = 5.74, p = 0.020), indicating that pain rating was significantly higher during pain than placebo treatment in HH participants (Fig 2B). The main effect for Treatment was near the significance level (F(1,49) = 3.85, p = 0.056), showing a trend towards a lower pain rating during placebo as compared to pain treatment.

The ANCOVA for distress ratings yielded a significant interaction of Hypnotizability x Condition (F(1,49) = 5.34, p = 0.025) and of Condition x Treatment x Hypnotizability (F(1,49) = 4.48, p = 0.039). Contrast analysis for the first effect indicated a significant distress reduction, in HH participants, during hypnosis compared to waking condition (p < 0.05, Fig 2C). The second effect indicated that HH participants, in both waking and hypnosis conditions, had a significant distress reduction for placebo versus pain treatment, while LH participants did not disclose significant changes between treatments and conditions (Fig 2D). No significant effects involving pain expectation were found for distress ratings.

### Startle eye-blink response

**Fig 3:**
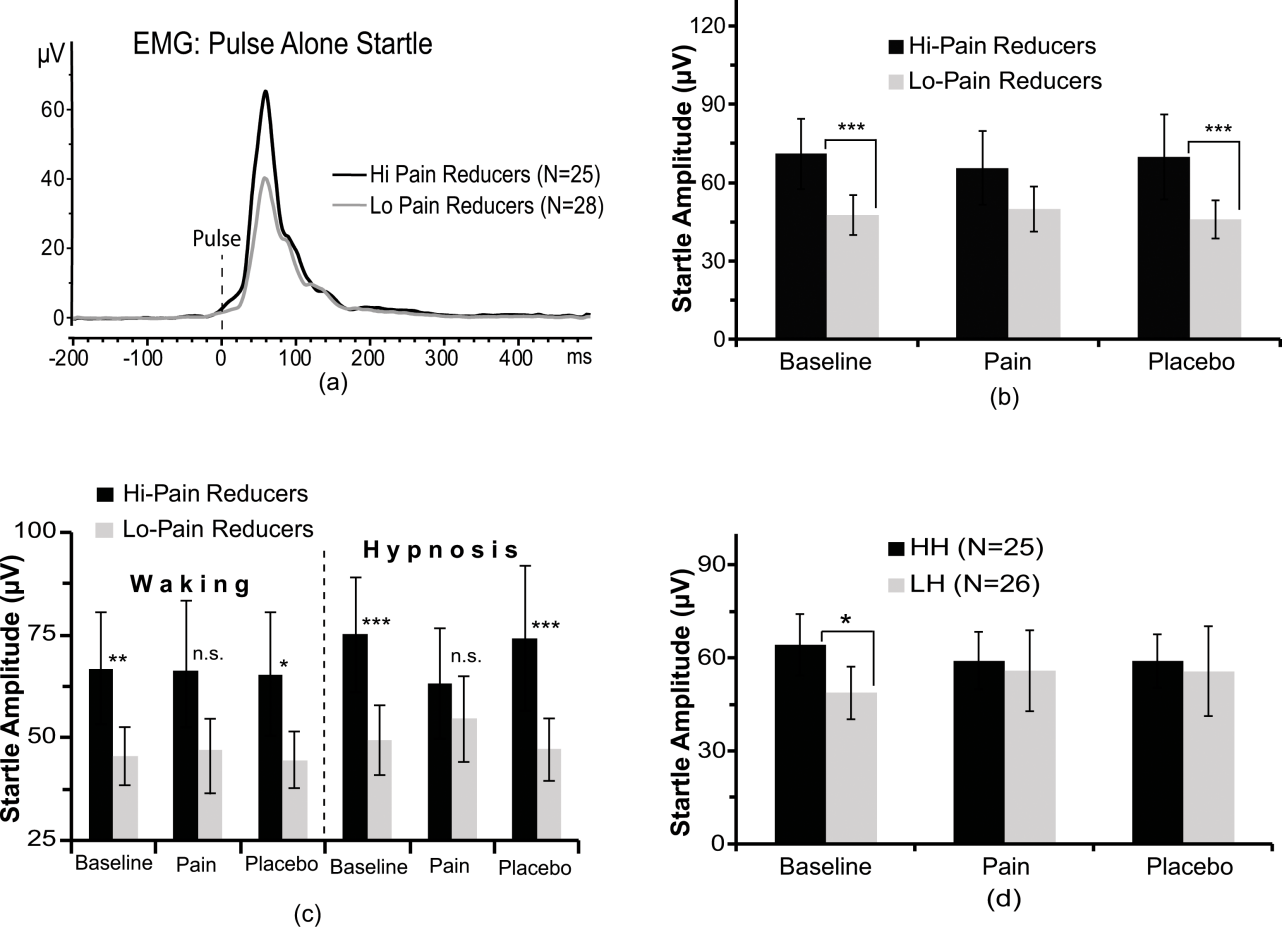
**Time course of a typical eye blink (EMG) startle response in high pain reducers and low pain reducers (panel a). Mean startle amplitudes and standard errors during Baseline, Pain, and Placebo treatments in high pain reducers and low pain reducers (panel b). Startle amplitude values across the three treatments in waking and hypnosis (panel c). Mean startle amplitudes across treatments in high and low hypnotizable participants (Panel d).** *p < 0.05, **p < 0.01, ***p < 0.001.

ANCOVA on peak amplitude of eye-blink response to PA startle probe yielded a significant main effect for Pain Reduction (F(1,49) = 8.68, p = .0049), which indicated a higher ASR in high pain reducers as compared to low pain reducers (Fig 3A). In addition, the interaction of Treatment x Pain Reduction and of Treatment x Pain Reduction x Condition were both significant (F(2,98) = 10.95, p < 0.0001, and F(2,98) = 11.30, p < 0.0001, respectively). Follow-up contrasts analysis for the first interaction indicated that during baseline and placebo treatments high pain reducers had a larger startle peak that low pain reducers (p < 0.001), while for pain treatment there were no significant differences between groups (Fig 3B). The second interaction effect indicated that pain reducers, in waking baseline and placebo treatments, had significantly higher eye-blink amplitude that low reducers (p < 0.05). However, during hypnosis this difference was more pronounced compared to waking condition (p < 0.001, Fig 3C). Finally, the interaction of Hypnotizability x Treatment was also significant (F(2,98) = 3.39, p < 0.05). This effect indicated that HH participants, during the baseline, had a larger ASR than LH ones. During both pain and placebo treatments this difference between hypnotizability groups was not significant (see Fig 3D). No other effects were significant.

### N100 and P200 peak amplitudes to PA startle

**Fig 4:**
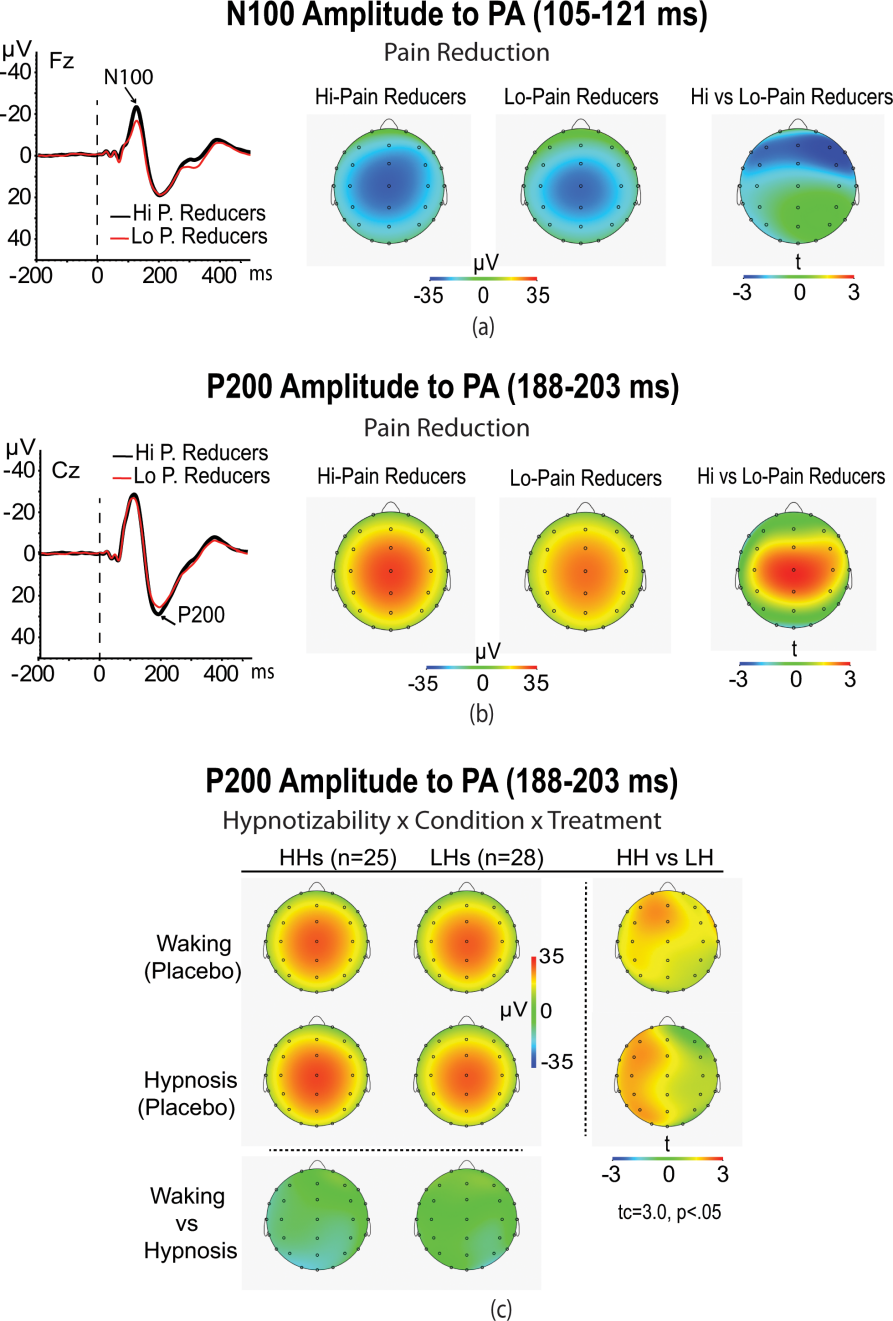
**ERPs of the most sensitive scalp sites (Fz and Cz) to auditory startle probes in high and low pain reducers (left panel a). Averaged scalp topography of N100 (panel a) and P200 wave (panel b) in high and low pain reducers** (t-test maps are reported on the right side)**. In panel (c) is shown the averaged scalp topography of the P200 peak in high hypnotizable (HHs) and low hypnotizable (HHs) participants during Placebo treatment in waking and hypnosis conditions.** t-Test map comparing HHs vs. LHs (maps on the right side) clearly shows for waking Placebo a larger P200 amplitude in the frontal left hemisphere, while for Placebo treatment during hypnosis this left-hemisphere difference was distributed from frontal to occipital sites (p < 0.05).

ANCOVA performed on N100 midline amplitudes scores elicited by PA stimuli yielded a significant main effect of Pain Reduction (F(1,49) = 4.78, p = 0.02). Follow-up contrasts indicated that high pain reducers had a larger N1 peak on frontal recordings (Fz) than low pain reducers (M = 25.1 μV, SD = 3.5 μV vs. M = 19.4 μV, SD = 1.9 μV, p < 0.05). This difference is displayed in Fig 4A.

ANCOVA performed on P200 midline amplitudes yielded a main effect for Pain Reduction (F(1,49) = 4.09, p = 0.049). Follow-up contrasts indicated that high pain reducers had a larger N1 peak on central recordings (Cz) than low pain reducers (M = 36.2 μV, SD = 2.1 μV vs. M = 32.5 μV, SD = 1.6 μV, p < 0.05; see Fig 4B).

The second order interaction of Hypnotizability x Condition and the third order interaction of Hypnotizability x Condition x Treatment were both significant (F(1,49) = 5.05, p = 0.029, and F(2,98) = 3.15, p = 0.044, respectively). Contrasts analysis indicated that, during placebo treatment in waking condition, the HH participants had a larger P200 wave than the LH ones (F(1,49) = 5.63, p = 0.022; M = 33.7 μV, SD = 5.7 μV vs. M = 25.5 μV, SD = 4.8 μV) while, for placebo treatment during hypnosis, this difference between hypnotizability groups was even more pronounced (F(1,49) = 8.01, p < 0.01; M = 35.5 μV, SD = 6.7 μV vs. M = 24.5 μV, SD = 4.1 μV; see Fig 4C). No other significant effects were found.

### LORETA source localizations and individual differences

To test for statistically significant differences in regional brain activation between groups, separate t-tests of high vs. low levels in Pain Reduction and Hypnotizability were performed on sLORETA waveforms derived from ERP waves.

**Fig 5:**
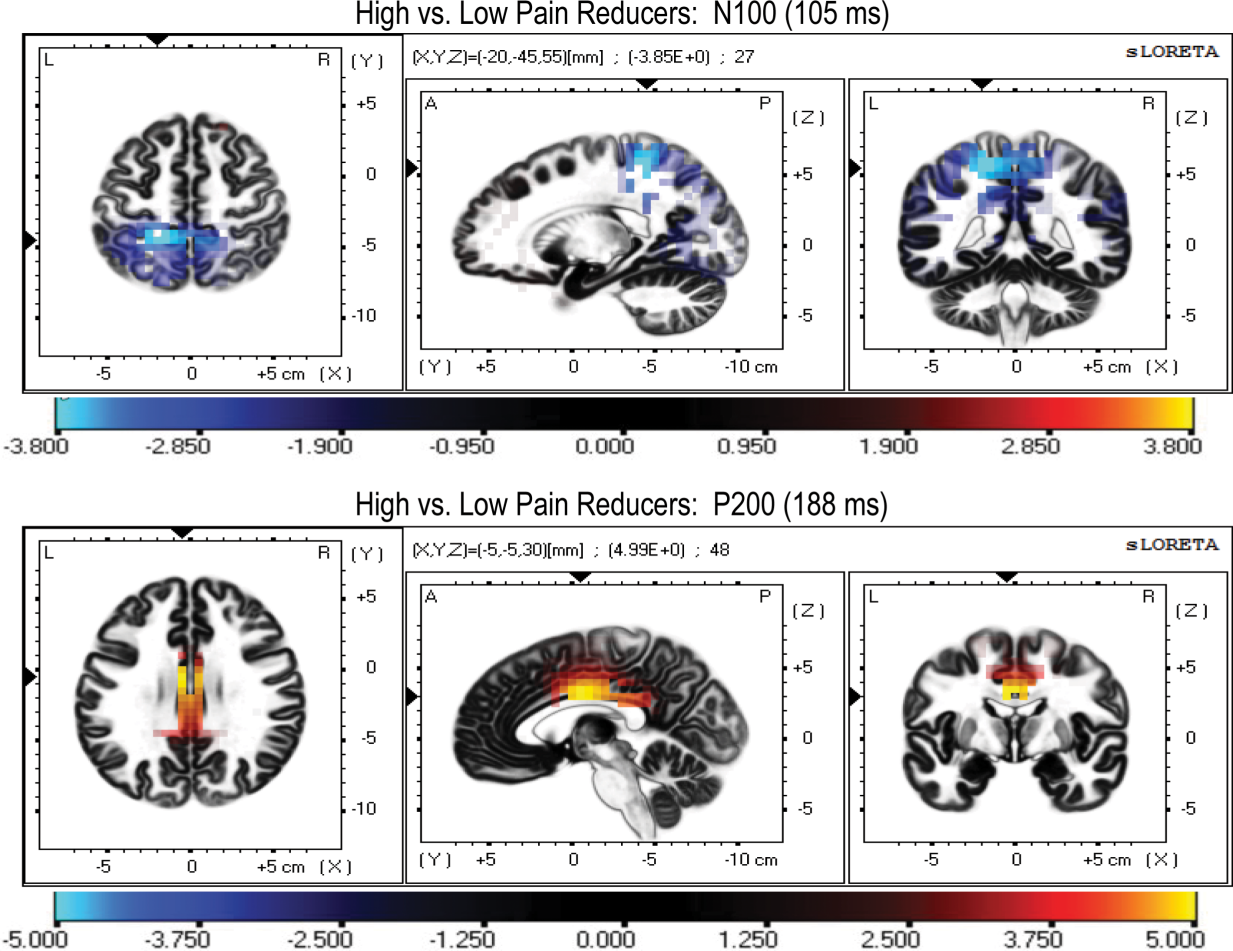
**LORETA parametric maps comparing N100 peak (top panel) and P200 peak (bottom panel) of the ERPs to auditory startle probes of high vs. low Pain reducers.** Note that high pain reducers showed a higher activation in the left paracentral lobule of the frontal lobe (BA5) and in the postcentral and sub-gyral gyri lobule of the parietal lobe (BA3 and BA40) at 105 ms (N100 peak). For the P200 peak, high pain reducers had a higher activation at 188 ms in the cingulate gyrus of the limbic lobe (BA24, BA23, and BA31); coordinates are shown in Table 2.

For Pain Reduction, we found that high pain reducers had a significantly higher activation at 105 ms (i.e., a maximal negative t value in the time window of N100) in the left paracentral lobule of the frontal lobe (BA5), the left postcentral gyrus of the parietal lobe (BA3), and in the sub-gyral of the parietal lobe (upper panel of Fig 5 and Table 2). High pain reducers also showed a greater activation at 188 ms (i.e., a maximal positive t value in the time window of P200 wave) in the cingulate gyrus of the limbic lobe (BA24, BA23, and BA31). These regions are mapped in the lower panel of Fig 5 and reported in the bottom of Table 2.

**Table 2:** MNI coordinates and Brodmann areas (BA) of statistically stronger cerebral activation in High Pain Reducers (N = 25) compared with Low Pain Reducers (N = 28) for the N100 (max BA at 105 ms) and P200 waves (max BA at 188 ms) elicited by pulse-alone startle.

ERP	X	Y	Z	t(max)*	BA	Lobe	Region	No. Voxels p<0.05
**N100**	-20	-45	55	-3.85	L5	Frontal	Paracentral Lobule	25
	-20	-40	60	-3.82	L3	Parietal	Postcentral Gyrus	5
	-25	-40	55	-3.69	L40	Parietal	Sub-Gyral	4
**P200**	-5	-5	30	4.99	L24	Limbic	Cingulate Gyrus	53
	0	-5	35	4.98	C24	Limbic	Cingulate Gyrus	11
	5	-5	35	4.84	R24	Limbic	Cingulate Gyrus	36
	-5	-15	30	4.80	L23	Limbic	Cingulate Gyrus	21
	0	-15	30	4.78	C23	Limbic	Cingulate Gyrus	6
	5	-15	30	4.80	R23	Limbic	Cingulate Gyrus	10
	-10	-25	40	4.35	L31	Limbic	Cingulate Gyrus	17
	0	-30	35	4.33	C31	Limbic	Cingulate Gyrus	10
	20	-30	45	4.35	R31	Limbic	Cingulate Gyrus	18

*t-crit. = 3.65, p < 0.05; t-crit. = 4.48, p < 0.01

Note: For N100 wave, a negative value of t indicates a higher CSD for High Pain Reducers than Low Pain Reducers. For P200 wave, a positive value of t indicates a higher CSD for High Pain Reducers than Low Pain Reducers.

**Fig 6:**
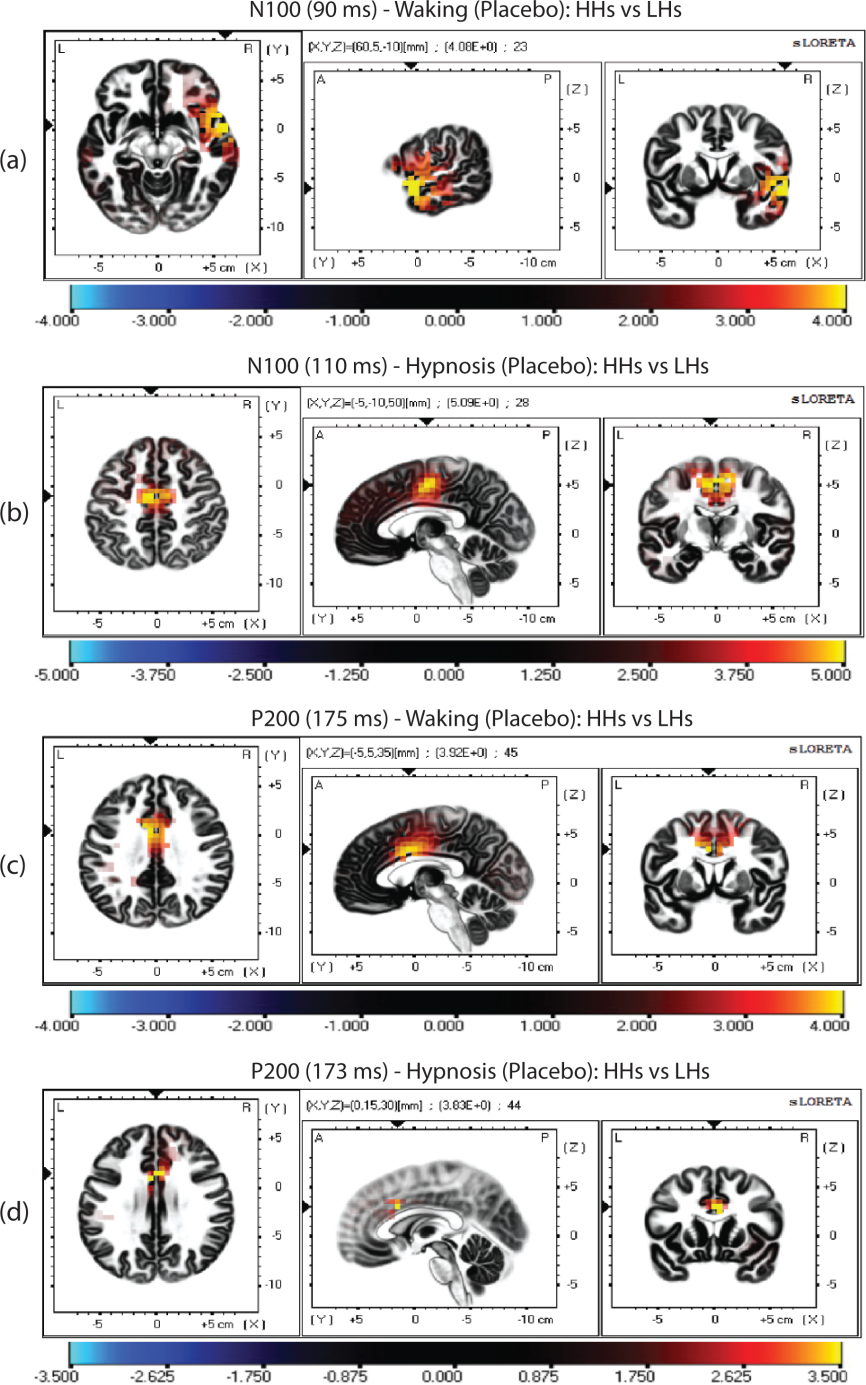
**LORETA parametric maps comparing N100 peak of high vs. low hypnotizable participants (HHs vs. LHs) in waking (a) and hypnosis conditions (b). Similar comparisons were done for the P200 peak in waking (c) and hypnosis (d).** For the N100 peak note that HHs compared to HHs during waking showed a lower activation in the right middle and superior temporal gyri (BA21, BA22, BA38) at the 91 ms time frame (N100 peak) while, during hypnosis, the maximal differences between hypnotizability groups were found at 110 ms in the cingulate gyrus (BA24), in the medial frontal gyrus (BA6), and paracentral lobule (BA31). For the P200 peak note that HHs compared to HHs during both waking and hypnosis conditions showed higher activations in the cingulate gyrus of the limbic lobe (BA24) at about a 175- ms time frame. Coordinates are shown in Table 3.

In terms of individual differences in Hypnotizability, we found that, during placebo treatment in waking condition, HH participants, compared to LH ones, had a significantly lower activity at 90 ms (N100 wave) in the middle and superior temporal gyri (BA21, BA22, BA38) of the right temporal lobe. Interestingly, during placebo treatment in hypnosis significant differences between hypnotizability groups were found at 110 ms in the cingulate gyrus (BA24), the medial frontal gyrus (BA6), and paracentral lobule (BA31). These regional differences are displayed in Fig 6A and 6B and upper section of Table 3. For the P200 wave, we found that during placebo in waking condition HH, compared to LH participants, had a significantly higher activity at 175 ms in the left and central cingulate gyrus (BA24), while during hypnosis these differences between hypnotizability groups were found at 173 ms in the central and right cingulate gyrus (BA24). These differences are shown in Fig 6C and 6D and in the lower section of Table 3.

**Table 3:** MNI coordinates and Brodmann areas (BA) of statistically lower cerebral activation for the N100, and higher activation for the P200 wave, in High Hypnotizable (HH, N = 25) compared to Low Hypnotizable participants (LH, N = 28) during Placebo treatment in Waking and Hypnosis conditions, as elicited by pulse-alone startle. The statistically stronger activation for the N100 (Waking: 91 ms, Hypnosis: 110 ms) and P200 (Waking: 175 ms, Hypnosis: 173 ms) is reported.

Placebo Analgesia	X	Y	Z	t(max)*	BA	Lobe	Region	No.Voxels p<0.05
**Waking**								
**N100 (91 ms)**	60	5	-10	4.08	R21	Temporal	Middle Temporal Gyrus	7
	60	5	-5	4.01	R22	Temporal	Superior Temporal Gyrus	10
	55	5	-10	4.00	R38	Temporal	Superior Temporal Gyrus	6
**Hypnosis**								
**N100 (110 ms)**	-5	-10	50	5.09	L24	Limbic	Cingulate Gyrus	11
	0	-5	45	4.70	C24	Limbic	Cingulate Gyrus	4
	10	-10	45	4.75	R24	Limbic	Cingulate Gyrus	8
	-5	-15	55	4.87	L6	Frontal	Medial Frontal Gyrus	7
	5	-10	55	5.00	R6	Frontal	Medial Frontal Gyrus	10
	-5	-15	50	4.87	L31	Frontal	Paracentral Lobule	4
**Waking**								
**P200 (175 ms)**	-5	-5	35	3.92	L24	Limbic	Cingulate Gyrus	11
	0	0	35	3.79	C24	Limbic	Cingulate Gyrus	3
**Hypnosis**								
**P200 (173 ms)**	0	15	30	3.82	C24	Limbic	Cingulate Gyrus	2
	5	15	30	3.67	R24	Limbic	Cingulate Gyrus	2

*Waking t-crit. = 3.94, p < 0.05; t-crit. = 4.33, p < 0.01; Hypnosis t-crit. = 3.61, p < 0.05; t-crit. = 4.30, p < 0.01

Note: For the N100 wave, a positive value of t indicates that HH show lower CSD than LH participants. For the P200 wave, a positive value of t indicates a higher CSD for HH than LH participants.

## Discussion

In the current study we found that higher pain expectation, rated before participants had experienced experimental pain for the first time (Fig 1A), was associated with higher levels of pain reduction through placebo treatment in waking condition (Table 1, and Fig 2A). Although we have not found another study reporting this relation, this effect is sometimes observed in a clinical context and can be reasonably understood if we assume that an inhibitory conservative mechanism is operating in order to fit with the expected painful event. In contrast, during hypnotic placebo analgesia the association of pain expectation with pain or distress reduction was weakened and not significant (Table 1). We conceived this result assuming that hypnosis disrupted the upper-mentioned significant associations found in waking condition. This result supports previous findings suggesting that placebo and hypnosis involve, at least to some extent, different processes of top-down regulation [19]. This observation also corroborates our previously reported pain-hypnosis ERP findings [31]

In terms of hypnotic susceptibility, our results are consistent with a number of previous reports showing that HH individuals are more responsive to suggestions for pain reduction [33, 78, 87, 122–126] than LH ones (Fig 2B). In addition, during hypnosis we found an enhanced distress reduction compared to the waking condition (Fig 2C).

In terms of eye-blink, N100, and P200 startle responses, we did not find significant differences between pain and placebo treatments compared to baseline. These lacking effects parallel Deuter and colleagues’ startle findings [73] and deserve further explanation. It has been reported that directing attention to the startle-eliciting stimulus can increase the startle response, while directing attention to a painful stimulus can reduce startle magnitude [127]. Attentional and emotional factors interact and may work in opposite directions, making the net effect on startle responsiveness difficult to predict [128]. If they are equal in size and point in opposite directions, no observable net effect would appear. This may have been the case for the EMG and ERP startle responses we detected across treatments. However, we also found that pain reducers reported significant enhancements of EMG, N100, and P200 amplitudes to the startle probe across all experimental conditions (Fig 3A, 3B, 3C and Fig 4). This difference between high and low pain reducers for the N100 was more frontally and, for the P200, more centrally distributed (see t maps in Fig 4A and 4B). These findings corroborate the hypothesis that relief of subjective painfulness is highly pleasant and rewarding [129, 130] and that less attentional resources were directed to the cold-pain stressor, making attention more direct towards the startle-eliciting stimulus. This indirect modulation in selective attention, as a consequence of pain reduction, may have enhanced startle and ERP responses [54, 127, 131–137]. Interestingly, these findings are in line with the observations suggesting that brain activity increases in the prefrontal cortex during both placebo [26, 27, 138–141] and hypnosis treatments for pain reduction [32–35]. The involvement of prefrontal cortex indicates that cognitive evaluation and attention are most likely to play an important role in pain relief. Overall, we found that during placebo treatment in waking the HH, compared to LH participants, had a significantly higher P200 wave across midline and frontal leads in the left hemisphere, while during placebo treatment in hypnosis this difference was spread throughout the left hemisphere (i.e., contralaterally to the side of painful stimulation; t maps Fig 4C). These differences between HH and LH groups could be due to the fact that the HH, but not LH, had significant pain/distress reductions to placebo treatment during waking and, even more pronounced during hypnosis. This could have enhanced saliency of the auditory stimulation and, thus, reoriented attention to the most significant alerting stimulus [137], a cognitive-control function attributed to the left frontal cortex [142]. In addition, during hypnosis the activation involved the whole left hemisphere including the occipital cortex. These results parallel original findings suggesting that waking placebo treatment is associated to an increased activity in the prefrontal cortex [26, 138–141, 143], while hypnotic placebo analgesia is associated to activity changes, other than in the prefrontal cortex, throughout the left-hemisphere including occipital cortex, which is concerned with mental imagery processing [32–34, 144].

Our interpretation of the present findings is also supported by previous reports showing that LH individuals, compared to the HH ones, usually possess weaker abilities to focus and sustain their attention as well as to pop out, from the environmental context, irrelevant stimuli and these differences are reflected in underlying brain dynamics [31, 61, 145]. This conclusion fits well with Horton and colleagues’ neuroimaging findings of a larger rostrum of corpus callosum in HH participants, compared to LH ones, and with studies indicating a more efficient mechanism of sensory gating in the former, rather than the latter [58, 60].

Our LORETA analysis of the N100 and P200 ERP waves disclosed that the left-paracentral lobule in the frontal lobe (BA5), the left-postcentral (BA3), and the left-sub-gyral in the parietal lobe (BA40) were significantly more activated in high pain reducers at 105 ms (N100 wave) than low pain reducers. In addition, these participants for the P200 wave had the maximal activation at 188 ms in the anterior cingulate (BA24) and posterior cingulate (BA23 and BA31) gyrus of the limbic lobe (see Fig 5 and Table 3). These regional findings, together with N100 and P200 ERP findings (Fig 4), indicated that pain reduction, in an early stage of stimulus-driven attentional processing, requires the activation of the left frontal and parietal lobes, whereas in a later stage of processing requires the activation of anterior and posterior cingulate gyres. This observation is corroborated by prior neuroimaging and animal research findings indicating that candidate structures for the modulation of startle amplitudes are the prefrontal cortex, caudate nucleus, cingulated cortex, and thalamus [40, 146–149].

In terms of individual differences in hypnotizabiliy, our LORETA analysis highlighted that in HH, compared to LH participants, waking placebo treatment produced an early decreased activity in the right middle and superior temporal gyrus (90 ms from tone onset, N100 wave; BA21, BA22, and BA38, see Fig 6A and Table 3). This effect was followed by a late increased activity in the ACC (175 ms, P200 wave; Fig 6C and Table 3). Hypnotic placebo analgesia in HH, compared to LH participants, disclosed an early (110 ms, N100 wave) decreased activity in the ACC (BA24), medial frontal gyrus (BA6), and left-paracentral lobule (BA31; see Fig 6B and Table 3). Hypnotic placebo analgesia, in a later processing stage (175 ms, P200 wave), showed an increased activity in the ACC (Fig 6C and 6D and Table 3). It is interesting to note that differences in cortical regional activity between waking and hypnosis placebo were found in an early processing stage from the startle probe onset. That is, waking placebo showed an earlier inhibition in the right temporal lobe, while hypnotic placebo an earlier inhibition (110 ms) in both frontal and ACC regions. In addition, in a late processing stage common to both waking placebo and hypnotic placebo analgesia was the enhanced activity in the ACC. Research has shown that these cortical regions are part of a pain responsive network (i.e., somatosensory cortex, ACC, insula, perigenual cortex, pre-supplementary motor cortex, thalamus, and prefrontal cortex) [141, 150–152] whose activity is modulated by the ongoing pain experience associated with hypnotic analgesia [20, 32, 33, 58, 153, 154]. Thus, we think that regional differences in electro cortical activity, found between hypnotizability groups, reflect the higher pain/distress reduction reported by HH participants. Considering that both the N100 and P200 waves are believed reliable measure of sensory gating [37, 155, 156], these findings can suggest a more efficient sensory gating in HH in comparison to LH participants, and indicate the important role of the right temporal-frontal lobe and ACC in the regulation of this function.

The present results extend current findings on hypnotic modulation of brain activity in a nonclinical sample [31, 33, 157, 158] and support the view that the effects of placebo and hypnosis on pain relief are, at least to some extent, separate processes [19, 27, 32–35]. However, it is important to underline that current source findings are purely speculative and must be considered with caution, since they were obtained using only 30 scalp electrodes and the modeling is based on a standard head model (instead of individual MRI data). With a weaken spatial resolution, there is a smaller chance that LORETA will be able to separate two closely spaced sources [159]. Another limitation of the current study is that our findings are restricted to women participants and, thus, cannot be generalized to men. Thus, further studies are necessary to validate our findings using an enhanced spatial resolution and by considering gender, attention, and heterogeneity of hypnotizability as potential factors influencing somatic and electrocortical startle responses during placebo analgesia in waking and hypnosis.

## Conclusion

The present findings are in line with prior reports that the placebo treatment in both waking and hypnosis condition can reduce pain and distress perception. Moreover, they show that hypnosis is not equal to common placebo in terms of brain activity, thus questioning the hypothesis that the pain reducing properties of hypnosis are just one form of placebo effect.

We found that higher pain expectation was associated with higher levels of pain reduction through placebo treatment in waking condition, but during hypnosis this association was under the significance level. We thought that this observation supports the hypothesis that placebo analgesia in waking and hypnosis reflects two different top-down processes [19].

Moreover, we have demonstrated that pain reduction induces an enhancement of EMG startle, a larger N100 wave at frontal sites, and P200 wave at central sites. These findings suggest that pain relief, being highly rewarding, makes that more processing capacity is available to process auditory startle probes. The validity of this explanation is also supported by our observation that in HH, compared to LH participants, placebo analgesia in waking condition enhances the activity in the prefrontal cortex [26, 27, 138–141] while, during placebo treatment in hypnosis, this difference involved the left frontal cortex and the posterior left hemisphere including occipital cortex, which is associated with mental imagery processing [32–34, 144].

Our LORETA analyses have highlighted that, in HH participants, waking placebo analgesia is characterized by an earlier decreased activity in the right temporal cortex (N100 wave), followed by a late increased activity in the ACC (P200 wave). During hypnosis, placebo analgesia is characterized by an earlier reduced activity in the medial frontal cortex and ACC (N100 wave), followed by a late increased activity in the ACC (P200 wave). These cortical regions are part of the previously described pain processing network [141, 150–152]. The present study extends original findings on hypnotic modulation of brain activity in a normal sample [31, 33, 157, 158] and shows that hypnotic placebo analgesia differs from common placebo analgesia in respect to pain experience and brain functioning.

## Supporting Information

S1 Appendix Participants (Section A in S1 Appendix), Pain threshold measures (Section B in S1 Appendix), and Suggestive treatment (Section C in S1 Appendix).
(PDF)
