# Supplementary material for: Hypnotizability and Placebo Analgesia in Waking and Hypnosis as Modulators of Auditory Startle Responses in Healthy Women: An ERP Study
Source: PLoS One. 2016 Aug 3;11(8):e0159135. doi: 10.1371/journal.pone.0159135 (PMC4972439; doi:10.1371/journal.pone.0159135)
Supplement: S1 Appendix — Participants (Section A in S1 Appendix), Pain threshold measures (Section B in S1 Appendix), and Suggestive treatment (Section C in S1 Appendix). (PDF) [file pone.0159135.s001.pdf]

**Hypnotizability and Placebo Analgesia in Waking and Hypnosis as modulators of Auditory Startle Responses in Healthy Women: An ERP Study**

Vilfredo De Pascalis, Paolo Scacchia

# SUPPLEMENTAL APPENDIX S1.

## Section A
## Participants

Only physically healthy participants were included in the study. Inclusion criterion demanded the absence of any lifetime history of significant psychiatric or neurologic disease, drug abuse, head trauma or loss of consciousness, treatment with antipsychotic medication, substance abuse or dependence use of amphetamine or cocaine (excluding caffeine and nicotine), and the absence of medical conditions that might interfere with pain sensitivity (e.g., high blood pressure, diabetes mellitus, asthma, heart diseases, frostbite, arthritis, Raynaud's syndrome, post-trauma to hands). The subjects were asked to refrain from smoking or drinking coffee for at least three hours before the EEG recording. Care was taken to ensure that participants had no information about their level of hypnotizability. Among selected participants, 13 were nondaily smokers. The decision to include all female subjects was informed by reports indicating that women are significantly more susceptible to hypnosis than men [1,2], although more recent research has demonstrated that gender differences can be rather small even when found [3]. In addition, in terms of gender differences on ASRs, findings indicate larger ASRs and weaker prepulse inhibition (PPI) in women compared to men [4,5]. Since previous research has demonstrated that inhibition of the startle response is reduced in luteal women compared to follicular women [6], participants who were in a menstrual period were invited for the EEG recordings between the 5th and 11th day after the onset of menses. Since there is no clear demonstration that hypnotizability changes across menstrual cycle phase, ASR and hypnotizability levels were evaluated in the late noon of the same day.

References

1. Page RA, Green JP (2007) An update on age, hypnotic suggestibility, and gender: a brief report. Am J Clin Hypn 49: 283-287.
2. Rudski JM, Marra LC, Graham KR (2004) Sex differences on the HGSHS:A. Int J Clin Exp Hypn 52: 39-46.
3. Cardeña E, Kallio S, Terhune D, Buratti S, Lööf A (2007) The effect of translation and sex on hypnotizability testing. Contemporary Hypnosis 24: 154-160.
4. Swerdlow NR, Caine SB, Braff DL, Geyer MA (1992) The neural substrates of sensorimotor gating of the startle reflex: a review of recent findings and their implications. J Psychopharmacol 6: 176-190.
5. Della Casa V, Hofer I, Weiner I, Feldon J (1998) The effects of smoking on acoustic prepulse inhibition in healthy men and women. Psychopharmacology (Berl) 137: 362-368.
6. Jovanovic T, Szilagyi S, Chakravorty S, Fiallos AM, Lewison BJ, et al. (2004) Menstrual cycle phase effects on prepulse inhibition of acoustic startle. Psychophysiology 41: 401-406.

## Section B
## Pain threshold measures

For this measure, each participant was required to tightly hold in the right hand, maintaining the same grip, a tin-plastic cup full of frozen water (CCT test, external surface temperature of -10 °C)

for a stimulation time of 3.7 minutes. After this time, participants had to rate their pain and distress sensation using the NRS (0 = no pain/distress, 10 = onset of pain sensation, and 100 = unbearable pain/distress). During the stimulation, they were also asked to indicate the instant in which they perceived the onset of a pain sensation (i.e., a rating of 10) by saying "now." A chronometer served to measure the time (sec) that each participant perceived the onset of pain. All subjects reached the pain threshold of 10 within a time interval ranging from 8 to 30 sec (M = 20.0 sec, SD = 9.6 sec), while the mean and standard deviation of subjective pain and distress ratings during the whole stimulation time of 3.4 min were respectively of M = 51.4, SD = 22.8, and M = 33.1, SD = 27.0.

**Section C**
**Suggestive treatment**
During the manipulation and pain treatments in waking and hypnosis, the following suggestion was given to each participant (pause of 1-2 sec are indicated as "..."):
"In a moment ... I will apply on the palm of your right hand the analgesic cream... ok? (the experimenter waits for the participant's response) ... Can I borrow your arm? (if yes, the experimenter ambiguously touches the arm of the participant to suggest a dissociative catalepsy) ... good …While I spread the analgesic cream (with a cotton swab), you may already start to feel some sensation in that hand ... and as you begin to feel this sensation, it means that the analgesic is starting to work ... now ... the effect of this analgesic could be like ... I do not know if you have ever forgotten the arm in a certain position and realize ... that the hand is beginning to tingle ... numbness ... insensitive ... you can feel a growing perception of tingling, numbness in that hand ... well ... and that hand absorbs more the active ingredient of the analgesic drug, more becomes tingling, numb, insensitive, ... you can start to feel as if that hand ... is not yours ... now ... the feeling that causes this analgesic is just like a hand tingling, numb, insensitive ... as if that hand is detached from your body, now. Usually the first effects of analgesic begin immediately after the application of the ointment and it needs 8 minutes to get its maximum effect. Now that I've applied the analgesic cream on that hand, I will make another measurement just when the analgesic drug has achieved its maximum effect ... ok? (the experimenter waits for the participant's response)... Well ... and as the hand continues to absorb the analgesic cream, what do you begin to feel, more tingling, numb, insensitive … the palm or the fingers of that hand? ... (participant's response: fingers or palm). Well ... how would you describe that sensation? (tingling, numb, insensitive, other) and as that/those palm/fingers is/are becoming more tingling/numb/insensitive/other and it spreads also into the palm/fingers … now … as you stand here and that hand stands there, you can wait the 8 minutes necessary for the drug to achieve the maximum analgesic effect ... so that the hand can become increasingly numb, tingling, insensitive ... as if it is detached from your body ... (after a wait of about 3 minutes). Good ... and while the analgesic effect continues to spread throughout that hand, making it increasingly numb ... now ... in a moment I'll ask you to hold with that hand a thin plastic cup full of frozen water ... and after some time, that hand may feel just a little discomfort or become even insensitive. It may be that the hand is already tingling, numb, and insensitive ... as if it is detached from your body ... so that it can perceive a mild, easily bearable discomfort or only the pressure of the hand on the glass. Now that we have reached the 8 minutes necessary for the drug to achieve the maximum analgesic effect ... what do you feel, more tingling, numb, and insensitive in the palm or fingers of that hand? ... (subject response: fingers or palm). Well ... how would you describe that sensation (tingling, numb, insensitive, other)? And as that/those palm/fingers is/are becoming more tingling/numb/insensitive/other and it spreads also into the palm/fingers …. now [ ... ]"
